# LncRNA H19-rich extracellular vesicles derived from gastric cancer stem cells facilitate tumorigenicity and metastasis via mediating intratumor communication network

**DOI:** 10.1186/s12967-023-04055-0

**Published:** 2023-04-01

**Authors:** Hongying Zhao, Rongke Jiang, Chunmei Zhang, Zhijing Feng, Xue Wang

**Affiliations:** 1 Department of Oncology, Xuzhou City Cancer Hospital, Xuzhou Third People’s Hospital, Xuzhou Hospital Affiliated to Jiangsu University, No. 131 Huancheng Road, Gulou District, Xuzhou, 221000 People’s Republic of China; 2 Jiangsu University, Zhenjiang, 212013 People’s Republic of China

**Keywords:** Gastric cancer, Cancer stem cells, Extracellular vesicles, LncRNA H19, YAP, CDX2, Intratumor communication network

## Abstract

**Background:**

Extracellular vesicles (EVs) transport biologically active molecules, and represent a recently identified way of intercellular communication. Recent evidence has also reported that EVs shed by cancer stem cells (CSCs) make a significant contribution to carcinogenesis and metastasis. Here, this study aims to explore the possible molecular mechanism of CSCs-EVs in gastric cancer (GC) by mediating intratumor communication network.

**Methods:**

CSCs and non-stem cancer cells (NSCCs) were sorted from GC cells, and EVs were isolated from CSCs. H19 was knocked down in CSCs, and CSCs-EVs or CSCs-EVs containing shRNA-H19 (CSCs-EVs-sh-H19) were co-cultured with NSCCs, followed by evaluation of the malignant behaviors and stemness of NSCCs. Mouse models of GC were established and injected with CSCs-EVs from sh-H19-treated NSCCs in vivo.

**Results:**

CSCs had notable self-renewal and tumorigenic capacity compared with NSCCs. CSCs promoted the malignant behaviors of NSCCs and expression of stemness marker proteins through secretion of EVs. Inhibited secretion of CSCs-EVs curtailed the tumorigenicity and metastasis of NSCCs in vivo. H19 could be delivered by CSCs-EVs into NSCCs. H19 promoted the malignant behaviors of NSCCs and stemness marker protein expression in vitro along with tumorigenicity and liver metastasis in vivo, which was mechanistically associated with activation of the YAP/CDX2 signaling axis.

**Conclusion:**

Taken together, the present study points to the importance of a novel regulatory axis H19/YAP/CDX2 in carcinogenic and metastatic potential of CSCs-EVs in GC, which may be potential targets for anticancer therapy.

**Supplementary Information:**

The online version contains supplementary material available at 10.1186/s12967-023-04055-0.

## Introduction

Cancer stem cells (CSCs), the subpopulation of cancer cells, have the capability to drive tumor initiation, progression, metastasis, drug resistance, and recurrence, especially gastric cancer (GC) [1–3] This capability of CSCs is closely associated with their secretion of a large number of extracellular vesicles (EVs) with different cargos, including proteins, lipids, RNA, ssDNA, and dsDNA [4]. Thus, a better understanding of the molecular mechanisms of CSCs-derived EVs (CSCs-EVs) involved in the initiation and progression of GC may allow the development of therapeutic alternatives.

Long non-coding RNAs (lncRNAs) represent noncoding RNAs of over 200 nucleotides sequences, which have been extensively highlighted as therapeutic targets for malignancies due to their genome-wide expression in various tissues and the tissue specific expression characteristics [5]. H19 is a lncRNA firstly described as an oncofetal transcript, serving as a biomarker for cancers and a promising therapeutic target in human disorders [6]. H19 is shown to be overexpressed in GC tissues, and this overexpression relates to advanced pathological tumor stage and pathological tumor node metastasis stage; however, knockdown of H19 decreases GC cell invasion [7]. Meanwhile, another study has found that H19 depends on miR-675 to enhance the malignant phenotypes of GC AGS cells [8].

Moreover, previous results have shown that H19 knockdown inhibits activation of Yes-associated protein (YAP) by promoting YAP phosphorylation [9]. YAP is a transcriptional coactivator augmenting cell proliferation, stem cell maintenance and tissue homeostasis; upregulation of YAP enhances GC growth, progression and therapy-resistance [10]. Accumulating studies have demonstrated a positive correlation between YAP and caudal type homeobox transcription factor 2 (CDX2) expression where overexpression of YAP activates the expression of CDX2 whereas reduced expression of YAP inhibits the expression of CDX2 [11, 12]. Ectopically expressed CDX2 significantly promotes cell migration and invasion in GC AGS and MKN45 cells [13]. Although H19 positively correlates to YAP, the underlying mechanism of this interaction in GC remain unknown, and the association between H19 and CDX2 requires further investigation. Thus, the aim of the present study was to identify the functional relevance of H19 with the YAP/CDX2 axis in GC. The underlying mechanisms of H19-mediated YAP/CDX2 axis in the progression of GC was examined in order to provide novel insights into potential targets for the treatment of GC.

## Materials and methods

### Cell lines

Human GC cell lines NCI-N87 (CRL-5822, ATCC, Manassas, VA), HGC-27 (TCHu 22, Cell Bank of the Chinese Academy of Sciences, Shanghai, China) and MKN45 (CC-Y1358, EK-Bioscience Co., Ltd., Shanghai, China) and human embryonic kidney cell line HEK-293 T (CRL-3216, ATCC) were cultured in RPMI-1640 containing 10% fetal bovine serum (FBS) and 1% penicillin–streptomycin in a 5% CO_2_ incubator at 37 °C. Human normal gastric mucosal epithelial cell line GES-1 (CC-Y1572, EK-Bioscience) was cultured with Dulbecco's modified Eagle’s medium (DMEM). The medium was replaced every 24 h, digested with 0.25% trypsin every 72 h and passaged.

### Flow cytometric sorting

Single cell suspension of GC cells was blocked with phosphate-buffered saline (PBS) containing 10% FBS on ice for 15 min, and centrifuged at 1200*g* for 5 min. Cell pellet was then resuspended and incubated in staining buffer on ice with the following antibodies for 30 min to identify subsets of CSCs and non-stem cancer cells (NSCCs) in GC cells. The antibodies included CD44-fluorescein isothiocyanate (FITC) mouse monoclonal antibody (L178, 347943; BD Biosciences, San Diego, CA), and CD24-PE mouse monoclonal antibody (ML5, 560991; BD Biosciences), 20 μL of which was added to 10^6^ cells. Then, cells were stained with 2 ng/mL 4′,6-diamidino-2-phenylindole (DAPI; Sigma) and analyzed on a BD FACSAria™ III cell sorter (BD Biosciences) and FlowJo software (version 7.2.4, Tree Star Inc., Ashland, OR).

### Sphere formation assay

GC cells (1000 cells per well) were seeded in ultra-low attached 6-well plate in the sphere medium (3471, Corning, New York) and cultured in DMEM-F12 medium (A4192001, Thermo Fisher Scientific) supplemented with growth additive (S003K, Thermo Fisher Scientific) containing 1 μg/mL of hydrocortisone, 4 ng/mL of heparin, 20 ng/mL of human epidermal growth factor (EGF), 20 ng/mL basic fibroblast growth factor (bFGF) and 1% penicillin–streptomycin. Cells were incubated at 37 °C with 5% CO_2_ for 2 weeks or until the spheres formed and reached above 150 μm. The spheres were then imaged, and the diameter and number of spheres were determined using ImageJ software.

### Western blot

Total protein was extracted from cells or tissues, separated by sodium dodecyl sulfate–polyacrylamide gel electrophoresis (SDS-PAGE) and electroblotted onto membranes. The membrane was blocked using 5% bovine serum albumin (BSA) and underwent overnight incubation with primary antibodies (Additional file 1: Table S1) at 4 °C. The next day, the membrane was incubated with horseradish peroxidase (HRP)-labeled secondary antibody goat anti-rabbit/goat anti-mouse IgG (BA1054/BA1056, 1: 10,000, Boster Biological Technology Co. Ltd., Wuhan, Hubei, China). Enhanced chemiluminescence reagent (AR1172, Boster) was used to visualize the protein bands, which were quantified using ImageJ software [14].

### Isolation and identification of EVs

EVs were isolated from CSCs or NSCCs by the differential ultracentrifugation-based method as previously described [15]. The isolated EVs were then observed under a transmission electron microscopy (TEM; JEM-2000EX, JEOL, Tokyo, Japan). Briefly, 20 µL EV suspension was placed onto a 200-mesh carbon-coated grid for 3 min. followed by eliminating the excess fluid from the grid with a filter paper. The grid was allowed to dry at room temperature for 1 min. After that, EVs were counterstained with 30 μL of 3% phosphotungstic acid solution (pH 6.8) at room temperature for 5 min, dried using a filament lamp and photographed by TEM. Nanoparticle Tracking Analyzer (NS300, Malvern Instruments, Malvern, UK) was adopted to detect size distribution of EVs. Furthermore, Western blot was conducted to examine the EV surface marker proteins (CD9 [mouse, sc-13118, 1: 1000, Santa Cruz], CD81 [mouse, 66866-1-Ig, 1: 1000, Proteintech, Wuhan, China], and Alix [rabbit, 12422–1-AP, 1: 2000, Proteintech]) as well as non-EV marker proteins (HSP90 [rabbit, 4877S, 1: 1000, CST] and histone H3) [16].

### Uptake of EVs by GC cells

Cells cultured on four-well chamber slides were fixed using 4% paraformaldehyde for 15 min, and permeabilized using 0.5% Triton-X 100 for 20 min. For EV tracking, EVs were labeled using PKH26 red fluorescent dye (MINI26, Sigma) and incubated at room temperature for 5 min. The labeled EVs were resuspended in basic medium and incubated with the sorted NSCCs at 37 °C for 12 h. F-actin was stained using phalloidin-FITC (green, P5282, Sigma), and DAPI was used to label nuclei. Cy3-(miR-15b-3p inhibitor/inhibitor-negative control [NC]/mimics/NC) were synthesized, as well as purified by RiboBio Co. (China). A IX53 confocal laser scanning microscope (CLSM; LSM 510 META, Carl Zeiss AG, Oberkochen, Germany) was used to capture images.

### Cell counting kit-8 (CCK-8) assay

GC cells were seeded into 96-well plates at a density of 8 × 10^3^ cells/well and cultured in an incubator. Each well was added with 10 μL CCK-8 solution at specific time points (24, 48 and 72 h), and incubated in a 37 °C incubator for 1 h. Subsequently, the optical density (OD) values at 450 nm were determined by an Epoch microplate reader (Bio-Tek, Winooski, VT).

### Transwell assay

Cells (2.5 × 10^4^, 100 μL) were seeded into the upper chamber of Transwell that was pre-coated with 50 μL Matrigel (354234, BD Biosciences). Then 500 μL medium containing 10% FBS was placed in the lower chamber of Transwell. After 24 h, the invaded cells were stained with 0.1% crystal violet and counted in five randomly selected fields under an inverted microscope (IXplore Pro, Olympus, Japan). For cell migration measurement, Matrigel coating was not included and the remaining steps were consistent with cell invasion experiment.

### Lentiviral transduction

HGC-27 cells were seeded in 6-well plates at 2 × 10^5^ cell/mL for 24 h and transduced with lentivirus (Sigma; a titer of 10^8^ TU/mL) carrying short hairpin RNA (sh-) Rab27a and the control sh-NC. After 72 h, the medium was replaced with medium containing 4 μg/mL puromycin, and cell culture continued for at least 14 days. Puromycin-resistant cells were amplified in medium containing 2 μg/mL puromycin for 9 days and then transferred to puromycin-free medium to obtain HGC-27 cells with stable knockdown of Rab27a. shRNA sequences are detailed in Additional file 1: Table S2.

Using Lipofectamine 2000 reagent (11,668–019, Invitrogen, Thermo Fisher Scientific), GC cells at passage 3 were transfected with plasmids (Guangzhou RiboBio Co., Ltd., Guangzhou, Guangdong China) of overexpression (oe)-CDX2 (constructed by pEXP-RB-Mam [R11091.1, RiboBio] plasmid vector), oe-NC, sh-H19-1 (lnc3160303052832), sh-H19-2 (lnc3180507021406) and sh-NC (lnc3N0000001-1–5). After 48 h, RNA and protein were extracted for subsequent experiments.

### In vitro experimental protocols

CSCs were transduced with sh-NC, sh-H19-1 and sh-H19-2. NSCCs were treated with PBS, CSCs-EVs, CSCs-EVs-sh-NC, CSCs-EVs-sh-H19, dimethyl sulphoxide (DMSO), Verteporfin (YAP inhibitor, 10 µg/mL for 24 h; HY-B0146, MedChem Express), PBS + DMSO, CSCs-EVs + DMSO, CSCs-EVs + Verteporfin, oe-NC, oe-CDX2, CSCs-EVs-sh-NC + oe-NC, and CSCs-EVs-sh-H19 + oe-NC and CSCs-EVs-sh-H19 + oe-CDX2. After 24 h of pre-treatment with Verteporfin or the control DMSO, NSCCs were co-cultured with 1 μg EVs for 24 h [17]. After 48 h of transfection with oe-NC or oe-CDX2, NSCCs were co-cultured with 1 μg EVs for 24 h.

### RT-qPCR

Total RNA was extracted with TRIzol reagent (Invitrogen, Thermo Fisher Scientific), the concentration and purity of which were determined using NanoDrop2000 micro-UV spectrophotometer (1011U, NanoDrop, Thermo Fisher Scientific). The RNA was reverse-transcribed into cDNA with PrimeScript RT reagent Kit (RR047A, Takara, Japan). RT-qPCR was followed using Fast SYBR Green PCR Kit (RR820A, Takara) and the ABI PRISM 7300 RT-PCR System (Applied Biosystems). As normalized to GAPDH (Additional file 1: Table S3), the fold changes were calculated using the 2^−ΔΔCt^ method [18].

### Immunofluorescence

Cells were fixed with 4% formaldehyde for 15 min and then permeabilized with 0.1% Triton X-100 (P1080, Solarbio, Beijing, China) for 30 min. Afterwards, the cells were blocked with 5% BSA and probed with diluted primary antibodies to CDX2 (mouse, sc-393572, 1: 200, Santa Cruz), YAP (rabbit, ab52771, 1: 200, Abcam, Cambridge, UK), or active YAP (rabbit, ab205270, 1: 500, Abcam) overnight at 4 °C. The next day, the cells were re-probed with Alexa568-conjugated secondary antibody goat anti-mouse (A-21124, 1: 500, Invitrogen, Thermo Fisher Scientific) and Alexa488-conjugated goat anti-rabbit (A-11070, 1: 500, Invitrogen, Thermo Fisher Scientific) at 37 °C for 1 h. Thereafter, the cells were incubated with Alexa568-conjugated phalloidin (A12380, 1: 500; Invitrogen, Thermo Fisher Scientific) for 30 min and DAPI in the dark for 20 min and observed under a IX53 fluorescence microscope in five randomly selected fields.

### Establishment of mouse models

A total of 80 BALB/c nude mice (aged 4–5 weeks; weighing 18–22 g; Animal Experiment Center of Xuzhou Medical University, Jiangsu, China) were housed individually in the SPF laboratory at 25–27 °C and 45–50% humidity under a 12-h light/dark cycle for one week acclimatization. Mice were fasted for 12 h before administration, and given ad libitum access to water at other times. The current study was approved by the Animal Ethics Committee of Xuzhou City Cancer Hospital.

For the establishment of subcutaneous tumor models, 1 × 10^7^ CSCs were injected into the back of nude mice. Sixteen mice were inoculated with CSCs and NSCCs (n = 8 mice for each treatment). Tumor growth was measured using a Vernier caliper every week and calculated using the formula: V = (W^2^ × L)/2, where W indicates width and L indicates length. Seven weeks later, mice were euthanized, with tumors dissected, photographed and weighed.

For the establishment of orthotopic tumor models, 32 mice were inoculated with the mixture of CSCs sorted from sh-NC- and sh-Rab27a-transduced HGC-27 cells, treated with DMSO or Nexinhib20 (a specific inhibitor of Rab27a; 1 μM for 4 h; SML1919, Sigma) with the NSCCs sorted from HGC-27 cells without any treatment into the gastric subserosa.

In addition, 32 mice were implanted with NSCCs (sorted from HGC-27 cells without any treatment) treated with PBS, CSCs-EVs (100 μg EVs in 100 μL PBS via tail vein injection), CSCs-EVs-sh-NC and CSCs-EVs-sh-H19 in situ into the gastric subserosa (n = 8 mice for each treatment). Administration was conducted twice every week. Seven weeks later, mice were euthanized, with tumor tissues and liver tissues dissected, photographed, weighed, frozen in liquid nitrogen and stored at − 80 °C.

### H&E staining

H&E staining was used for histological observation as previously described [19]. Tumor and liver tissues of mice were fixed, paraffin-embedded and sectioned (5 μm). Sections were stained with hematoxylin (Solarbio), and counterstained with eosin. Next, the sections were observed under a XP-330 optical microscope (Shanghai Bingyu Optical Instrument Co., Ltd., Shanghai, China).

### Immunohistochemical staining

GC tissue specimens were cut into 5-μm-thick sections. The sections were blocked with normal goat serum and immunostained with primary antibodies to active YAP, CDX2 and Rab27a overnight at 4 ºC, and then with biotin-labeled secondary antibody goat anti-rabbit or goat anti-mouse (BM3894 or BM3895, 1: 500, Boster). Next, the sections were incubated with 50 μL streptomyces anti-biotin–peroxidase solution at room temperature for 10 min and developed with DAB. Following counterstaining, dehydration, clearing and mounting, the sections were observed with a microscope. Primary antibody was substituted by PBS. Positive cells were brown-yellow and the sum of the integrated optical density (IOD) was analyzed using Image-ProPlus6.0 [20].

### Statistical analysis

Unpaired *t*-test, and one-way ANOVA were utilized to calculate statistical significance for two-group, muti-group and time-based data. All results processed using SPSS 21.0 software and GraphPad Prism 8.0 were presented as mean ± standard deviation. *p* < 0.05 suggests statistically significant difference. Levene's test was used to test the homogeneity of variance of the data. Dunnett* t*-test and LSD *t*-test were used for data with homogeneity of variance while Dunnett's T3 test for data with defect variances.

## Results

### CSCs isolated from GC cells have significant tumorigenic capacity

**Fig. 1:**
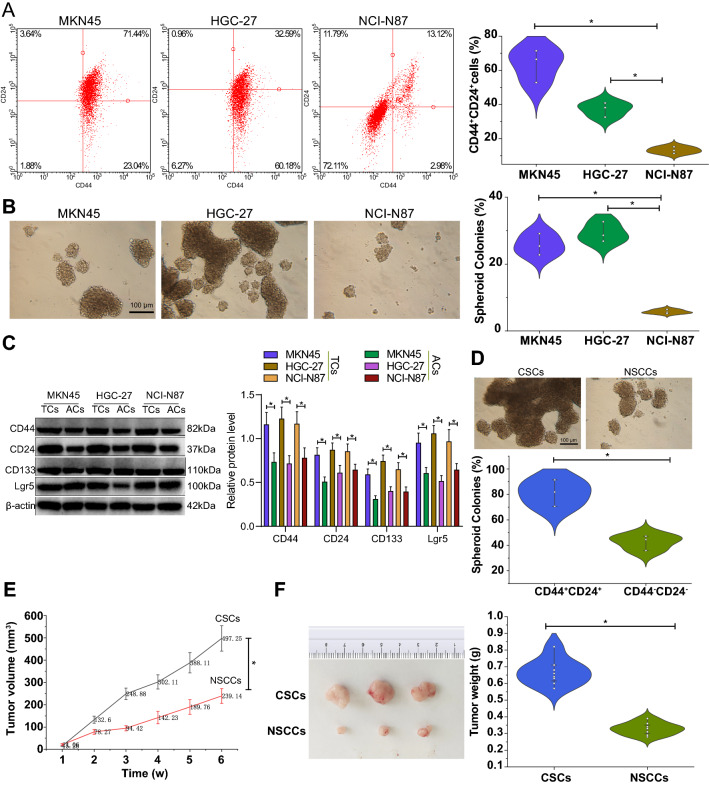
Isolation and identification of CSCs and NSCCs. **A** The expression of stem cell surface markers CD44 and CD24 in human GC cell lines detected by flow cytometry. The II and III quadrants indicate CD44^+^, and the III quadrant indicates CD44^+^ and CD24^+^. **B** Colony formation of GC cell lines in serum-free medium. **C** Western blots and quantification analysis of the stemness marker proteins CD44, CD24, CD133, and Lgr5 in the tumorsphere and adherent cells of each GC cell line. TCs indicate tumorsphere cells and ACs indicate adherent cells. **D** Sphere-forming ability of CD44^+^CD24^+^ and CD44^−^CD24^−^ cells in HGC-27 cells. **E** Tumor volume of mice inoculated with CSCs (CD44^+^CD24^+^) and NSCCs (CD44^−^CD24^−^) at 1–7 weeks. **F** Tumor weight of mice inoculated with CSCs (CD44^+^CD24^+^) and NSCCs (CD44^−^CD24^−^). Cell experiments were conducted three times independently. n = 8 mice for each treatment. *p < 0.05

In this study, we first examined the expression of candidate surface markers for CSCs in the human GC cell lines MKN45, HGC-27, and NCI-N87 by flow cytometry. According to the current results, the CSCs with CD44^+^CD24^+^ phenotype had significantly higher tumorigenic potential compared with non-tumorigenic cells. The CD44^+^CD24^+^ cells exhibit obvious self-renewal properties of stem cell [21]. Therefore, we selected CD44 and CD24 as tumor cell stemness markers in GC and detected their expression. Detection results showed high proportion of CD44^+^CD24^+^ cells in MKN45 and HGC-27 cells, up to 71.44% and 32.59%, respectively, while the positive rate of CD44^+^CD24^+^ cells in NCI-N87 cells was only 13.12% (Fig. 1A).

The above GC cell lines were cultured with serum-free medium, and after 2 weeks, MKN45, HGC-27 and NCI-N87 cells formed spherical colonies, with MKN45 and HGC-27 having more spherical colonies (Fig. 1B). Evidence has shown that the tumorsphere- and colony-forming ability of tumor cells is regulated by the stemness of tumor cells and the enhanced stemness of CSCs is often closely related to the abnormal expression of CD44, CD24, CD133, and Lgr5 [22–24]. Hence, we detected the protein expression of stemness markers CD44, CD24, CD133 and Lgr5 in tumorsphere cells and adherent GC cells by Western blot. The results presented an increase of the protein expression of CD44, CD24, CD133 and Lgr5 in tumorsphere cells compared to adherent cells (Fig. 1C). These results indicate that HGC-27 cells have stronger stemness characteristics compared to MKN45 and NCI-N87 cells. Therefore, HGC-27 cells were selected for the subsequent experiments.

HGC-27 cells were sorted to obtain the CSCs (CD44^+^CD24^+^) and NSCCs (CD44^−^CD24^−^). Sphere formation assay results indicated more spheres formed in CD44^+^CD24^+^ cells than those in CD44^−^CD24^−^ cells (Fig. 1D). CSCs (CD44^+^CD24^+^) and NSCCs (CD44^−^CD24^−^) were then inoculated into the abdomen of nude mice, and the tumor weight and volume were found to be increased in the presence of CSCs compared with NSCCs (Fig. 1E, F).

The above results show the successful isolation of CSCs and NSCCs in GC cell lines, and that CSCs have obvious self-renewal and tumorigenic capacity.

### EVs mediate the communication between CSCs and NSCCs in GC

**Fig. 2:**
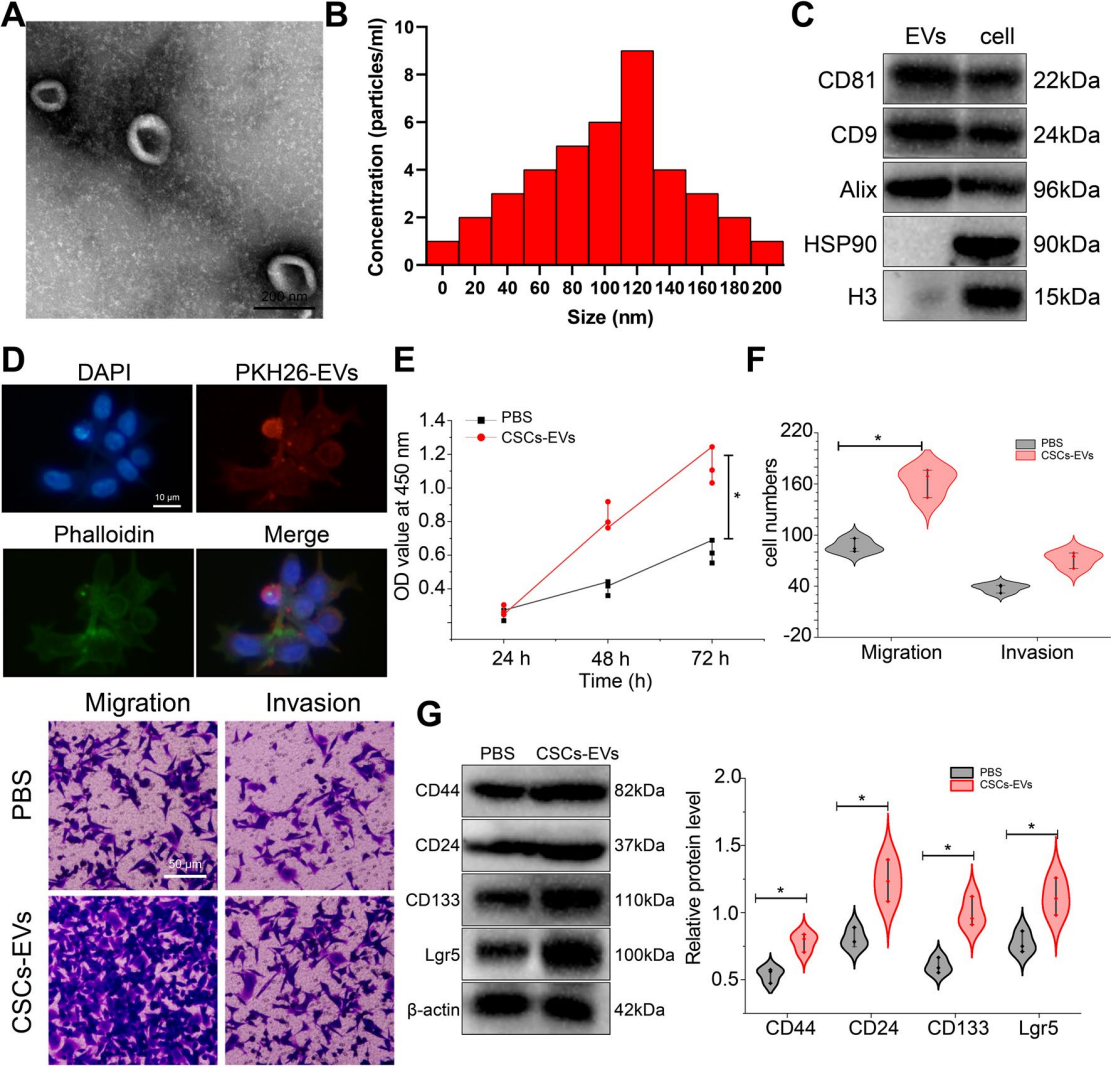
CSCs-EVs elevate the malignant behaviors of NSCCs and stemness marker protein expression. **A** Morphology of CSCs-EVs observed under a TEM. **B** NTA of the particle size of CSCs-EVs. **C** Western blot of the EV positive markers CD81, CD9, Alix, and the negative markers HSP90 and histone H3 in CSCs-EVs. **D** Immunofluorescence staining of the uptake of CSCs-EVs by NSCCs. **E** Viability of NSCCs co-cultured with CSCs-EVs measured by CCK-8 assay. **F** Migration and invasion of NSCCs co-cultured with CSCs-EVs measured by Transwell assay. **G** Western blot of the stemness marker proteins CD44, CD24, CD133, and Lgr5 in NSCCs co-cultured with CSCs-EVs. Cell experiments were conducted three times independently. *p < 0.05

To explore whether there was EV-mediated communication network between CSCs and NSCCs in GC, we first extracted EVs from CSCs cells. Before applying the extracted EVs for subsequent experiments, we analyzed the basic properties and particle size distribution of EVs by TEM and NTA. EVs isolated from CSCs showed cup-shaped morphology with lipid bilayer structure. The outer layer was the deeply stained area of the bilayer lipid membrane, while the inner layer was the unevenly lightly stained area, with typical morphological characteristics of EVs (Fig. 2A). In addition, the particle size of most EVs ranged 124.1 ± 4.5 nm (Fig. 2B). Further, Western blot was conducted to determine the expression of EV marker proteins. EV marker proteins CD81, CD9, and Alix were expressed in CSCs-EVs, while HSP90 and histone H3 were hardly expressed in the EVs (Fig. 2C). The above results suggest that the extracted EVs conform to the basic characteristics of EVs and can be used for subsequent experiments. Under a fluorescence microscope, PKH26-labeled CSCs-EVs showed red fluorescence in the NSCC cytoplasm (Fig. 2D), which showed that CSCs-EVs could be internalized by NSCCs.

The aforementioned data allowed us to speculate whether CSCs-EVs internalized by NSCCs can endow NSCCs with CSCs-like characteristics. For verification, we detected the effect of CSCs-EVs on the proliferation and migration of NSCCs. The results of CCK-8 and Transwell assays showed that the proliferation and migration of NSCCs co-cultured with CSCs-EVs were enhanced (Fig. 2E, F). In addition, the protein expression of CD44, CD24, CD133 and Lgr5 was elevated in the NSCCs co-cultured with CSCs-EVs (Fig. 2G).

Taken together, EVs can mediate the communication between CSCs and NSCCs in GC. Besides, CSCs-EVs promote the malignant behaviors of NSCCs and stemness marker protein expression.

### Knockdown of Rab27a inhibits the secretion of EVs, thereby curtailing tumorigenicity and metastasis of GC NSCCs in vivo

**Fig. 3:**
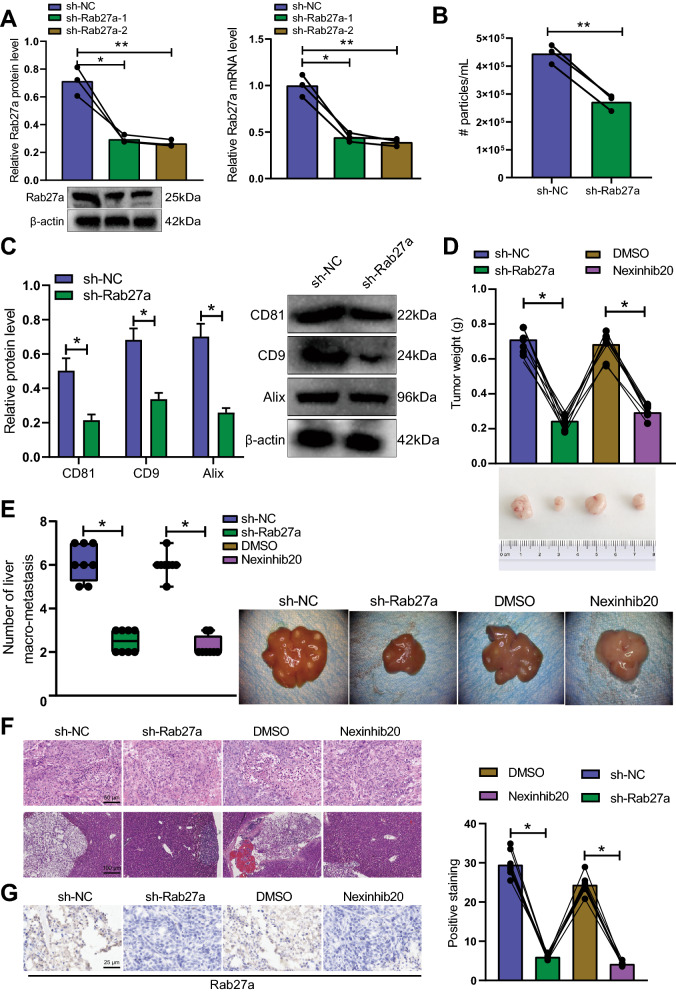
Rab27a knockdown disrupts secretion of EVs and suppresses the tumorigenicity and metastasis of GC NSCCs in vivo. **A** Rab27a expression in HGC-27 cells treated with sh-Rab27a-1 or sh-sh-Rab27a-2 measured by Western blot and RT-qPCR. **B** NTA of the number of EVs released by HGC-27 cells treated with sh-Rab27a. **C** Western blots and quantification analysis of EV positive marker proteins CD81, CD9, and Alix in HGC-27 cells treated with sh-Rab27a (the remaining protein expression in response to decreased expression of CD81, CD9 and Alix in the presence of sh-Rab27a was calculated with sh-NC as the reference). **D** Representative images showing tumors in mice and tumor weight of mice treated with sh-Rab27a or Nexinhib20. **E** Representative images of liver tissues and the quantitative analysis of liver metastases in mice treated with sh-Rab27a or Nexinhib20. **F** H&E staining of tumor tissues and liver tissues of mice treated with sh-Rab27a or Nexinhib20. The tumor tissue is in the dotted box. **G** Positive expression of Rab27a protein in tumor tissues of mice treated with sh-Rab27a or Nexinhib20 detected by immunohistochemistry. Cell experiments were conducted three times independently. n = 8 mice for each treatment. *p < 0.05, **p < 0.01

Rab27a is a small GTPase involved in the exocytosis of vesicles of endosomal origin, and knockdown of Rab27a inhibited the secretion of EVs [25, 26]. In order to identify the possible role of EVs in the cancer cells, we performed Rab27a knockdown in cells. The effect of EVs on the tumorigenicity and metastasis of NSCCs in vivo was the subsequent focus of this study. Western blot and RT-qPCR data suggested a decline of Rab27a expression in HGC-27 cells treated with sh-Rab27a-1 or sh-sh-Rab27a-2 (Fig. 3A), with sh-Rab27a-2 showing a better knockdown efficiency. Therefore, sh-Rab27a-2 (sh-Rab27a) was used for the subsequent experiments.

Furthermore, the number of EVs released by the cells treated with sh-Rab27a was reduced (Fig. 3B). Protein expression of EV marker proteins CD81, CD9 and Alix [27] was downregulated in the presence of sh-Rab27a (Fig. 3C). These data demonstrate that the secretion of EVs was inhibited after Rab27a knockdown.

Tumor volume and weight were reduced in the mice treated with sh-Rab27a or Nexinhib20 [28] (Fig. 3D). In addition, there were few tumor nodules in liver tissues of mice treated with sh-Rab27a or Nexinhib20 (Fig. 3E). H&E staining data suggested decreased number of GC cells with liver metastasis in response to sh-Rab27a or Nexinhib20 (Fig. 3F). Meanwhile, immunohistochemical staining results revealed that Rab27a expression was downregulated in the tumor tissues of the sh-Rab27a- or Nexinhib20-treated mice (Fig. 3G).

Thus, knockdown of Rab27a retards the tumorigenicity and metastasis of GC NSCCs in vivo by curtailing the secretion of EVs.

### H19 delivered by CSCs-EVs facilitates the malignant behaviors of NSCCs and elevates the expression of stemness marker proteins

**Fig. 4:**
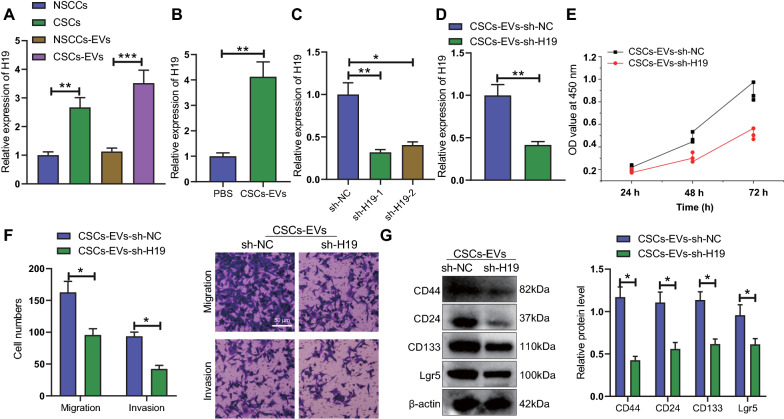
H19 delivered by CSCs-EVs enhances the malignant behaviors of NSCCs and upregulates the expression of stemness marker proteins. **A** H19 expression in NSCCs, CSCs and their EVs detected by RT-qPCR. **B** H19 expression in NSCCs co-cultured with CSCs-EVs detected by RT-qPCR. **C** Knockdown efficiency of H19 gene in CSCs detected by RT-qPCR. **D** H19 expression in NSCCs co-cultured with CSCs-EVs-sh-H19 detected by RT-qPCR. **E** Viability of NSCCs co-cultured with CSCs-EVs-sh-H19 determined by CCK-8 assay. **F** Migration and invasion of NSCCs co-cultured with CSCs-EVs-sh-H19 determined by Transwell assay. **G** Western blot of the stemness marker proteins CD44, CD24, CD133, and Lgr5 in NSCCs co-cultured with CSCs-EVs-sh-H19. Cell experiments were conducted three times independently. *p < 0.05, **p < 0.01, ***p < 0.001

We then aimed to explore the mechanism of CSCs-EVs affecting the biological function of NSCCs. H19 expression was elevated in HGC-27 cells compared with GES-1 cells (Additional file 2: Fig. S1A), and in CSCs and CSCs-EVs relative to NSCCs and NSCCs-EVs (Fig. 4A). In the co-culture system of NSCCs with CSCs-EVs, the expression of H19 was upregulated (Fig. 4B), which showed that CSCs-EVs can deliver H19 into NSCCs.

H19 was knocked down in CSCs and sh-H19-1 (sh-H19) with better knockdown efficiency was used for subsequent experiments (Fig. 4C). In the NSCCs, CSCs-EVs-sh-H19 led to reduced H19 expression (Fig. 4D). In addition, the proliferation and migration of NSCCs were suppressed upon co-culture with CSCs-EVs-sh-H19 (Fig. 4E, F). Western blot results confirmed that the protein expression of CD44, CD24, CD133 and Lgr5 was reduced in NSCCs co-cultured with CSCs-EVs-sh-H19 (Fig. 4G).

Overall, these data demonstrate that H19 delivered by CSCs-EVs facilitates the malignant behaviors of NSCCs and increases the expression of stemness marker proteins.

### H19 delivered by CSCs-EVs promotes YAP activation in NSCCs and thus increases CDX2 expression

YAP is highly expressed in GC and associated with the survival and migration of GC cells; H19 can induce YAP activation [9, 29]. Therefore, we speculated that H19 may promote YAP activation in GC.

**Fig. 5:**
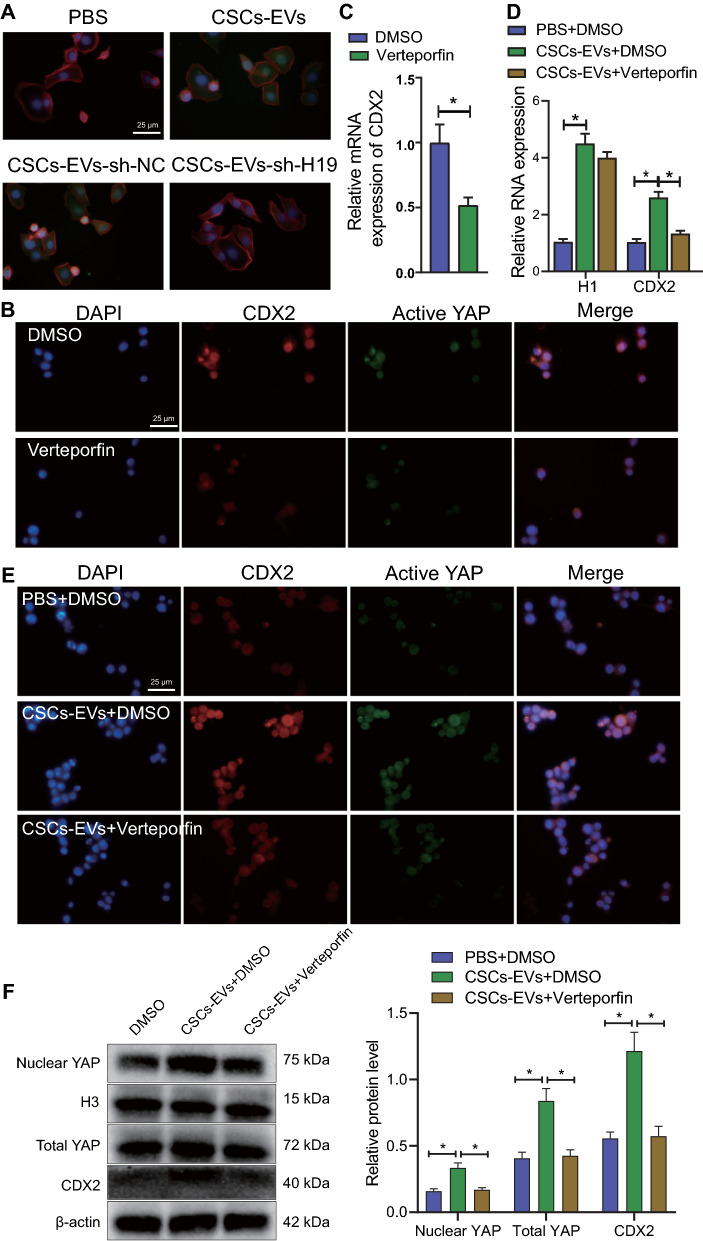
H19 delivered by CSCs-EVs induces YAP activation and augments CDX2 expression in NSCCs. **A** Expression of active YAP in the nucleus of NSCCs co-cultured with CSCs-EVs or CSCs-EVs-sh-H19 detected by immunofluorescence staining. **B** Expression of YAP and CDX2 in the nucleus of NSCCs treated with Verteporfin detected by immunofluorescence staining. **C** mRNA expression of CDX2 in NSCCs treated with Verteporfin determined by RT-qPCR. **D** H19 and CDX2 expression in NSCCs treated with CSCs-EVs + DMSO or CSCs-EVs + Verteporfin detected by RT-qPCR. **E** Expression of active YAP and CDX2 in the nucleus of NSCCs treated with CSCs-EVs + DMSO or CSCs-EVs + Verteporfin detected by immunofluorescence staining. **F** Western blots and quantification analysis of nuclear YAP, total YAP and CDX2 proteins in NSCCs treated with CSCs-EVs + DMSO or CSCs-EVs + Verteporfin. The grayscale analysis of the relative nuclear YAP expression used H3 as the internal reference, and that of the total YAP protein expression and CDX2 protein expression used β-actin as the internal reference. Cell experiments were conducted three times independently. *p < 0.05, **p < 0.01

Immunofluorescence staining results showed enhanced red fluorescence in the nucleus of HGC-27 cells and increased YAP protein expression versus GES-1 cells (Additional file 2: Fig. S1B). Further, NSCCs were co-cultured CSCs-EVs presented evident green fluorescence in the nucleus and upregulated expression of active YAP and total YAP protein. However, these effects were opposite in the NSCCs co-cultured CSCs-EVs-sh-H19 (Fig. 5A, Additional file 3: Fig. S2). These results indicate that CSCs-EVs carrying H19 could promote YAP activation in NSCCs.

Studies have reported that Hippo-YAP signaling is important for the activation of nuclear transcription factor CDX2 expression in mouse embryos [30, 31]. Meanwhile, CDX2 is preferentially expressed in GC [32, 33]. Therefore, we further explored the relationship between YAP and CDX2 in GC. The mRNA and protein expression of CDX2 was found amplified in HGC-27 cells compared to GES-1 cells, as determined by RT-qPCR and Western blot (Additional file 2: Fig. S1C, D). In addition, both YAP and CDX2 expression was diminished in the nucleus of NSCCs treated with the YAP inhibitor Verteporfin [34] (Fig. 5B), and the mRNA expression of CDX2 was also reduced (Fig. 5C).

Based on the results of RT-qPCR, the expression of H19 and CDX2 was increased in NSCCs treated with CSCs-EVs + DMSO while in the presence of CSCs-EVs + Verteporfin, H19 expression showed no changes, in addition to downregulated CDX2 expression (Fig. 5D). Increased YAP and CDX2 expression was detected in the nucleus of NSCCs treated with CSCs-EVs + DMSO while opposite results were noted in response to CSCs-EVs + Verteporfin (Fig. 5E). Furthermore, we conducted Western blot to detect the nuclear YAP expression, total YAP expression and downstream protein CDX2 in NSCCs co-cultured with CSCs-EVs or combined with treatment with YAP inhibitor Verteporfin. The results showed an increase of nuclear YAP expression, total YAP expression and CDX2 expression in NSCCs co-cultured with CSCs-EVs while treatment with Verteporfin led to opposite results (Fig. 5F).

Collectively, H19 delivered by CSCs-EVs could promote YAP activation, thus activating CDX2 expression in NSCCs.

### H19 delivered by CSCs-EVs enhances the malignant behaviors of NSCCs by downregulating the YAP/CDX2 signaling axis

**Fig. 6:**
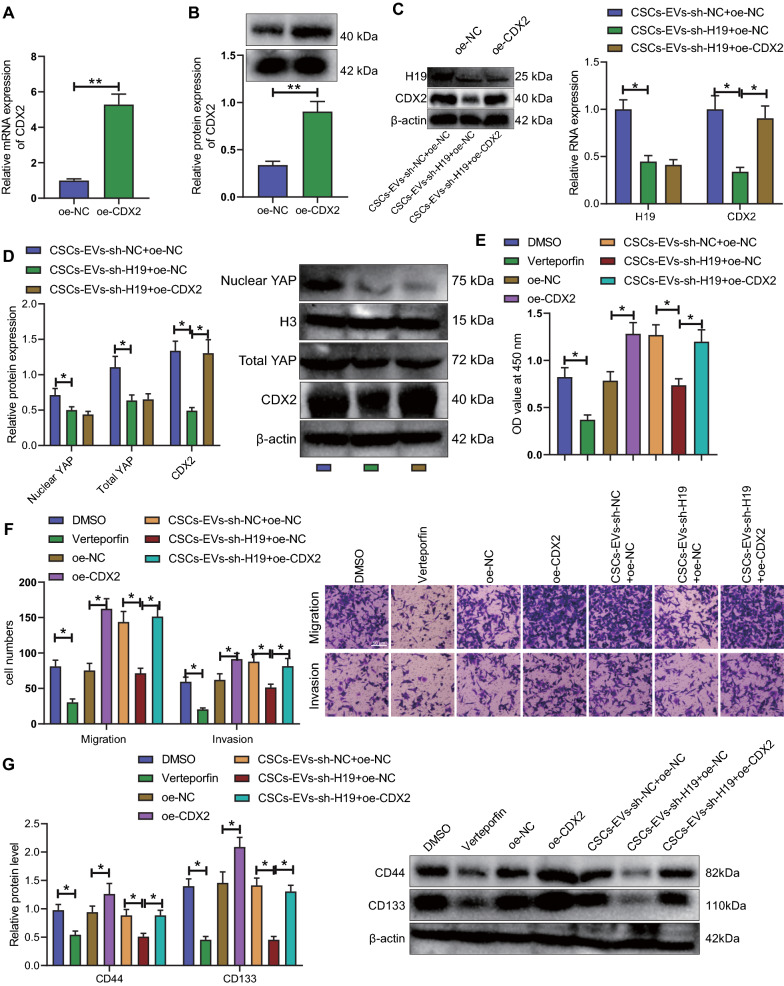
H19 delivered by CSCs-EVs facilitates the malignant behaviors of NSCCs by disrupting the YAP/CDX2 signaling axis. **A** mRNA expression of CDX2 in NSCCs treated with oe-CDX2 detected by RT-qPCR. **B** Western blot of CDX2 protein in NSCCs treated with oe-CDX2. **C** H19 expression and CDX2 mRNA expression in NSCCs analyzed by RT-qPCR. **D** Western blots and quantification analysis of the total YAP and nuclear YAP proteins and CDX2 protein in the NSCCs co-cultured with CSCs-EVs-sh-H19 + oe-NC or CSCs-EVs-sh-H19 + oe-CDX2. The grayscale analysis of the relative nuclear YAP expression used H3 as the internal reference, and that of the total YAP protein expression and CDX2 protein expression used β-actin as the internal reference. **E** Viability of NSCCs co-cultured with CSCs-EVs-sh-H19 + oe-NC or CSCs-EVs-sh-H19 + oe-CDX2 detected by CCK-8 assay. **F** Migration and invasion of NSCCs co-cultured with CSCs-EVs-sh-H19 + oe-NC or CSCs-EVs-sh-H19 + oe-CDX2 detected by Transwell assay. **G** Western blot of the stemness marker proteins CD44 and CD133 in the NSCCs co-cultured with CSCs-EVs-sh-H19 + oe-NC or CSCs-EVs-sh-H19 + oe-CDX2. Cell experiments were conducted three times independently. *p < 0.05, **p < 0.01

Having identified the correlation of H19 with YAP/CDX2 in NSCCs, we continued to explore the regulation of H19 on the biological function of NSCCs via mediation of the YAP/CDX2 signaling axis. The mRNA and protein expression of CDX2 was increased in the oe-CDX2-treated NSCCs (Fig. 6A, B). In the presence of CSCs-EVs-sh-H19 + oe-NC, a reduction was noted in the expression of H19 and mRNA and protein expression of CDX2 as well as the total and nuclear YAP protein expression; whereas, only mRNA and protein expression of CDX2 was augmented in NSCCs co-cultured with CSCs-EVs-sh-H19 + oe-CDX2 (Fig. 6C, D).

Verteporfin treatment obstructed the viability, migration and invasion of NSCCs while oe-CDX2 treatment led to opposite results. Following co-culture with CSCs-EVs-sh-H19 + oe-NC, NSCCs had suppressed viability, migration, and invasion. However, CSCs-EVs-sh-H19 + oe-CDX2 resulted in contrary results (Fig. 6E, F).

The protein expression of CD44 and CD133 was reduced in Verteporfin-treated NSCCs while oe-CDX2 induced opposite results. In response to co-culture with CSCs-EVs-sh-H19 + oe-NC, NSCCs suggested low expression of CD44 and CD133, which was reversed following co-culture with CSCs-EVs-sh-H19 + oe-CDX2 (Fig. 6G).

These lines of evidence demonstrate that inhibition of YAP activation curtails the malignant behaviors of NSCCs, while overexpression of CDX2 abolishes the effect. In addition, sh-H19 delivered by CSCs-EVs inactivates the YAP/CDX2 signaling axis, and impedes the malignant behaviors of NSCCs and the expression of stemness marker proteins.

### H19 delivered by CSCs-EVs activates the YAP/CDX2 signaling axis and promotes the tumorigenicity and metastasis of NSCCs in vivo

**Fig. 7:**
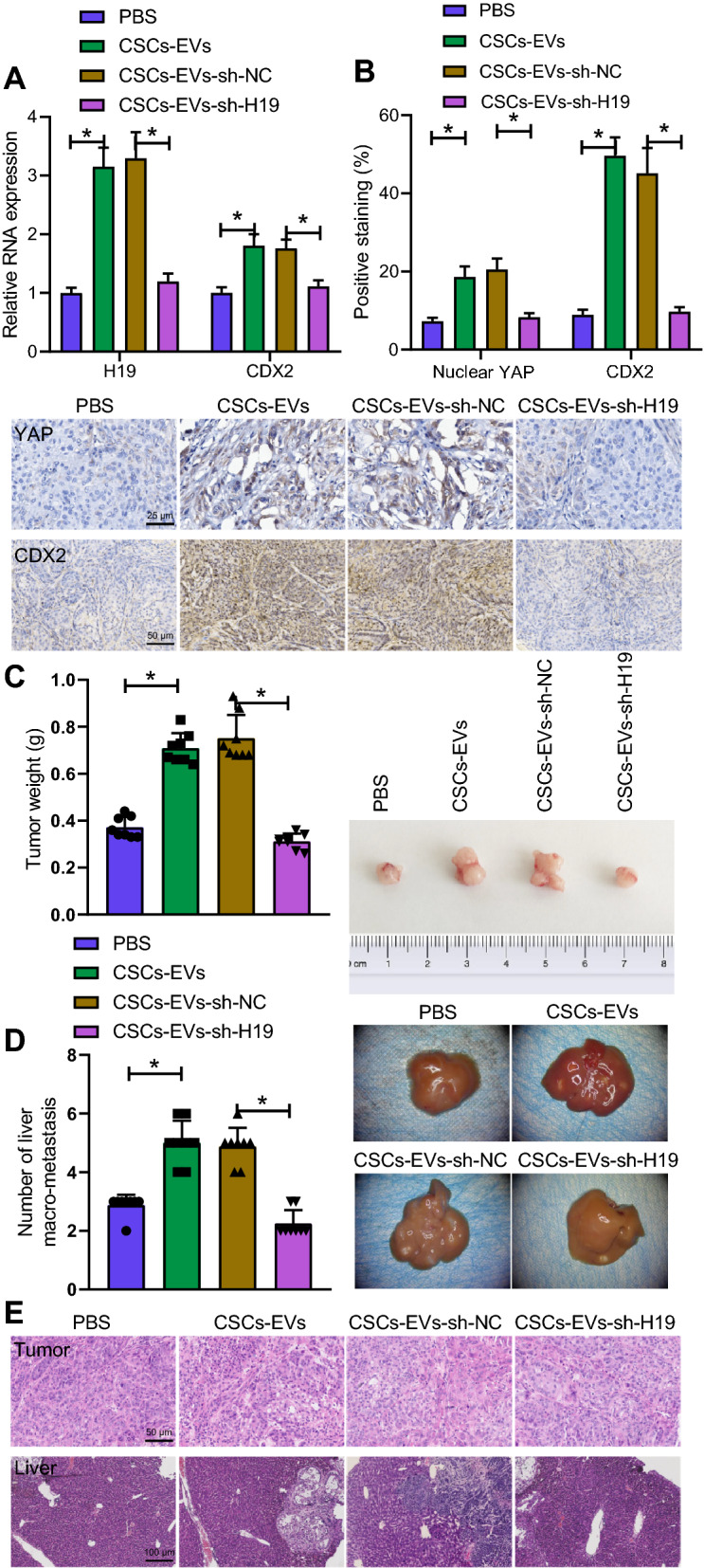
H19 delivered by CSCs-EVs activates the YAP/CDX2 signaling axis and facilitates the tumorigenicity and metastasis of NSCCs in vivo. **A** H19 and CDX2 expression in tumor tissues of mice treated with CSCs-EVs or CSCs-EVs-sh-H19 analyzed by RT-qPCR. **B** Positive expression of CDX2 and active YAP proteins in tumor tissues of mice treated with CSCs-EVs or CSCs-EVs-sh-H19 detected by immunohistochemistry. **C** Tumor weight of mice treated with CSCs-EVs or CSCs-EVs-sh-H19. D, Representative images of liver tissues and the quantitative analysis of liver metastases in mice treated with CSCs-EVs or CSCs-EVs-sh-H19. E, H&E staining of tumor tissues and liver tissues of mice treated with CSCs-EVs or CSCs-EVs-sh-H19. *p < 0.05. n = 8 mice for each treatment

Finally, we intended to analyze the effects of CSCs-EVs carrying H19 on the tumorigenicity and metastasis of GC cells in vivo. An elevation was noted in the mRNA expression of H19 and CDX2 in tumor tissues of CSCs-EVs-treated mice, while CSCs-EVs-sh-H19 decreased their expression (Fig. 7A). Additionally, the positive expression of nuclear YAP and CDX2 proteins was robustly induced in tumor tissues of CSCs-EVs-treated mice while an opposite effect was identified in the presence of sh-H19 (Fig. 7B).

Moreover, treatment with CSCs-EVs increased tumor weight of mice, while CSCs-EVs-sh-H19 reduced the weight (Fig. 7C). The tumor was large and the tumor nodules in liver tissues were increased upon treatment with CSCs-EVs, which was negated by CSCs-EVs-sh-H19 (Fig. 7D). The number of liver metastases was increased in response to treatment with CSCs-EVs but CSCs-EVs-sh-H19 caused converse results (Fig. 7E).

The above results serve to illustrate that H19 delivered by CSCs-EVs can activate the YAP/CDX2 signaling axis and accelerate tumorigenicity and metastasis of NSCCs in vivo.

## Discussion

CSCs have demonstrated critical roles in tumor development and progression via paracrine factors, including proteins, lipids, ssDNA, dsDNA, and RNA, delivered by EVs to recipient cells, orchestrating the cell–cell communication within tumor microenvironment [4]. In the present study, H19 was found highly expressed in the CSCs-EVs, which could transfer H19 into NSCCs. In these cells, H19 potentially activated YAP and increased CDX2 expression, therefore stimulating the malignant phenotypes of NSCCs, ultimately expediting GC progression.

Preferential communication occurs between pancreatic CSCs and pancreatic NSCCs, which can be mediated by EVs [25]. In the current study, EVs were found to mediate the communication between CSCs and NSCCs in GC. In addition, CSCs had obvious self-renewal and tumorigenic capacity relative to NSCCs, which is supported by published literature [35]. Besides, CSCs-EVs were demonstrated to be capable of promoting the malignant behaviors of NSCCs and stemness marker protein expression. However, the suppressed secretion of CSCs-EVs by Rab27a knockdown can curtail the tumorigenicity and metastasis of GC cells in vivo. Indeed, CSCs-EVs can contribute to tumor progression due to their involvement in tumor metastasis and the maintenance of stemness phenotype [36].

Subsequent results of the current study showed that silencing of H19 delivered by CSCs-EVs could reduce the malignant behaviors of NSCCs as well as decreasing the expression of stemness marker proteins in vitro. Also, the tumorigenicity and metastasis of NSCCs in vivo were suppressed upon knockdown of H19 encapsulated in CSCs-EVs. EVs can encapsulate lncRNAs and deliver them to the recipient cancer cells, where lncRNAs exerted tumor-promoting effects [37, 38]. H19 was found to be upregulated in GC tissues and cells while the downregulation of H19 suppresses the malignant phenotypes of GC cells in vitro and delays tumor growth in vivo [39]. Furthermore, H19 can induce β-catenin to transfer into the nucleus and activate the Wnt/β-catenin signaling, thus promoting the metastasis of GC cells in vitro and in vivo [40]. Meanwhile, H19 has been demonstrated to promote the stemness of colorectal CSCs [41].

This study further confirmed the underlying mechanism of H19 delivered by CSCs-EVs affecting the malignant behaviors of NSCCs and the expression of stemness marker proteins, which was associated with the activated YAP/CDX2 signaling axis. Consistently, H19 can induce YAP activation [9]. YAP is greatly elevated in GC cells and this elevation can drive the migration and survival of GC cells [42]. In addition, YAP contributes to promotion of stem cell maintenance [10]. Notably, downregulation of YAP results in a decline of CDX2 expression in GC cells [43]. CDX2 is abundant in GC tissues and cells, which augments the proliferative, migrating, and invasive properties of GC cells [32]. Altogether, these lines of evidence suggest a novel regulatory pathway of H19/YAP/CDX2 in GC which can serve as a potential target for cancer therapy.

## Conclusion

**Fig. 8:**
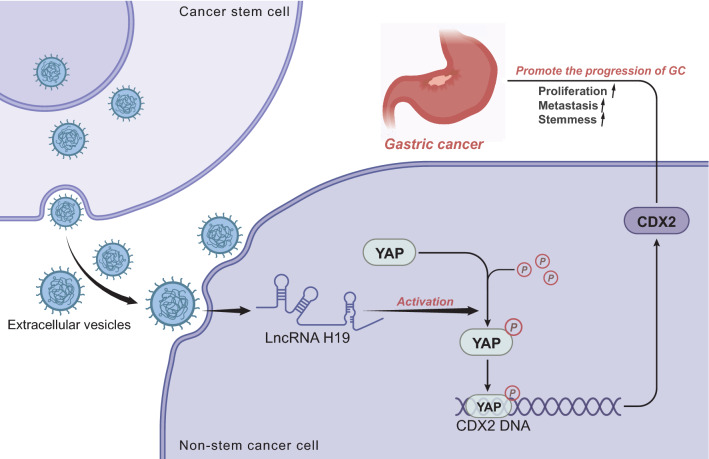
Schematic representation summarizing the role of CSCs-EVs carrying H19 in GC progression. CSCs-EVs can deliver H19 into NSCCs, where H19 activates the YAP/CDX2 signaling axis and facilitates the malignant phenotypes of NSCCs, ultimately promoting GC progression

In summary, the present study reveals that H19 transmitted by CSCs-EVs is a potential oncogene that activates the YAP/CDX2 signaling axis and promotes the growth and metastasis of GC NSCCs (Fig. 8). Though there are some limitations that the migratory and invasive activity of the cells can be further confirmed by examining cell proliferation- and invasion-related genes, these findings indicate the essential role of H19 transmitted by CSCs-EVs and its functional mechanisms in the malignant phenotypes of GC NSCCs. The findings of this study suggest that the H19/YAP/CDX2 axis may be a promising target for the treatment of GC.

## Supplementary Information

**Additional file 1: Table S1. **Details of the primary antibodies used for Western blot. **Table S2.** shRNA sequences. **Table S3.** Primer sequences for RT-qPCR.

**Additional file 2: Figure S1.** Detection of the expression of genes and proteins related to the H19/YAP/CDX2 signaling axis in GC cells. A, H19 expression in HGC-27 and GES-1 cells detected by RT-qPCR. B, Expression of YAP protein in the nucleus of HGC-27 cells detected by immunofluorescence staining. YAP staining is shown in red, and nuclear staining by DAPI is shown in blue. C, mRNA expression of CDX2 in HGC-27 cells detected by RT-qPCR. D, Western blot of CDX2 protein in HGC-27 cells. Cell experiments were conducted three times independently. *p < 0.05, **p < 0.01

**Additional file 3: Figure S2.** Total YAP expression in the nucleus of NSCCs detected by immunofluorescence staining. Cell experiments were conducted three times independently.

## Data Availability

The data supporting the fndings of this study could be obtained from the corresponding author.
