# Supplementary material for: LncRNA H19-rich extracellular vesicles derived from gastric cancer stem cells facilitate tumorigenicity and metastasis via mediating intratumor communication network
Source: J Transl Med. 2023 Apr 1;21:238. doi: 10.1186/s12967-023-04055-0 (PMC10067256; doi:10.1186/s12967-023-04055-0)
Supplement: Supplementary file 1 — Additional file 1: Table S1. Details of the primary antibodies used for Western blot. Table S2. shRNA sequences. Table S3. Primer sequences for RT-qPCR. [file 12967_2023_4055_MOESM1_ESM.docx]

**Table S1** Details of the primary antibodies used for Western blot

| Antibody | Dilution ratio | Cat. | Manufacturer | Country |
| --- | --- | --- | --- | --- |
| CD44 (rabbit) | 1: 5000 | ab51037 | Abcam | UK |
| CD24 (mouse) | 1: 1000 | sc-19585 | Santa Cruz | USA |
| CD133 (rabbit) | 1: 1000 | ab19898 | Abcam | UK |
| Lgr5 (rabbit) | 1: 1000 | ab75850 | Abcam | UK |
| active-YAP (rabbit) | 1: 1000 | ab205270 | Abcam | UK |
| YAP (rabbit) | 1: 5000 | ab52771 | Abcam | UK |
| H3 (rabbit) | 1: 2000 | ab201456 | Abcam | UK |
| RAB27a (mouse) | 1: 1000 | ab55667 | Abcam | UK |
| CDX2 (mouse) | 1: 1000 | sc-393572 | Santa Cruz | USA |
| β-actin (mouse) | 1: 10000 | AC004 | ABclonal | China |

**Table S2** shRNA sequences

| shRNA | Sequence |
| --- | --- |
| sh-Rab27a-1 | 5’-GCTGCCAATGGGACAAACATA-3’ |
| sh-Rab27a-2 | 5’-CGGATCAGTTAAGTGAAGAA-3’ |
| sh-NC | 5’-CTCGCTTGGGCGAGAGTAA-3’ |

**Table S3** Primer sequences for RT-qPCR

| Gene | Primer sequence |
| --- | --- |
| Rab27a | Forward: 5’-GCCATAGCACTCGCAGAGAA-3’ |
|  | Reverse: 5’-TGTCCACACACCGTTCCATT-3’ |
| H19 | Forward: 5’-GGACGTGACAAGCAGGACAT-3’ |
|  | Reverse: 5’-ATGTTGTGGGTTCTGGGAGC-3’ |
| CDX2 | Forward: 5’-CGGCAGCCAAGTGAAAAC-3’ |
|  | Reverse: 5’-GATGGTGATGTAGCGACTGTAGTG-3’ |
| GAPDH | Forward: 5’-CTCCTCCTGTTCGACAGTCAGC-3’ |
|  | Reverse: 5’-CCCAATACGACCAAATCCGTT-3’ |
